# Prevalence and Molecular Characterisation of *Cryptosporidium* spp. and *Giardia duodenalis* in Equines in Algeria

**DOI:** 10.1002/vms3.71083

**Published:** 2026-07-15

**Authors:** Nassiba Reghaissia, Yaseen Majid Salman Al‐Adilee, AbdElkarim Laatamna, Abdeldjalil Dahmane, Amine Berghiche, Houssem Samari, Samir Ansel, Yacine Laadjailia, Eleni Gentekaki, Martin Kváč, Nikola Holubová, Anastasios D. Tsaousis

**Affiliations:** ^1^ Laboratory of Sciences and Living Techniques Institute of Agronomic and Veterinary Sciences University of Souk Ahras Souk Ahras Algeria; ^2^ Laboratory of Exploration and Valorization of Steppic Ecosystems Faculty of Nature and Life Sciences University of Djelfa Djelfa Algeria; ^3^ Laboratory of Molecular and Evolutionary Parasitology School of Natural Sciences University of Kent Canterbury UK; ^4^ Department of Medical Microbiology College of Medicine Ninevah University Mosul Iraq; ^5^ Department of Veterinary Sciences Faculty of Exact Sciences Nature and Life Sciences University of Biskra Biskra Algeria; ^6^ Faculty of Sciences University of M'sila M'sila Algeria; ^7^ Faculty of Nature and Life Sciences University of khemis Meliana Khemis Meliana Algeria; ^8^ Department of Veterinary Medicine University of Nicosia School of Veterinary Medicine Nicosia Cyprus; ^9^ Biology Centre of the Academy of Sciences of the Czech Republic Institute of Parasitology České Budějovice Czech Republic; ^10^ Faculty of Agriculture and Technology University of South Bohemia in České Budějovice České Budějovice Czech Republic

**Keywords:** Algeria, *Cryptosporidium*, donkeys, genotyping, *Giardia duodenalis*, horses

## Abstract

**Background:**

Limited data are available on the epidemiology of *Cryptosporidium* spp. and *Giardia duodenalis* in equines in Algeria. No prior molecular data existed on giardiasis in this host population.

**Objectives:**

This cross‐sectional study aimed to determine the prevalence and molecular diversity of *Cryptosporidium* spp. and *G. duodenalis* in equines across multiple regions of Algeria, and to characterise species, subtypes and assemblages present.

**Methods:**

Between December 2021 and December 2022, 197 faecal samples were collected from 104 horses, 89 donkeys, 2 ponies, 1 zebra and 1 mule from 46 private farms, 2 equestrian centres and 1 zoological park in eastern and central Algeria. Detection used microscopy and PCR. *Cryptosporidium* species and subtypes were identified by sequence analysis of SSU rRNA and *gp60* genes. *G. duodenalis* was screened by probe‐based qPCR with genotyping attempted at the *tpi* and *bg* loci.

**Results:**

The overall molecular prevalence of *Cryptosporidium* was 34% (horses 31%, donkeys 38%), and *G. duodenalis* was 4.6% (horses 2.9%, donkeys 7%). Male equines had a significantly higher *Cryptosporidium* infection rate than females; no other epidemiological variable was significant for either parasite. Ten *Cryptosporidium* isolates were genotyped as four species: *C. parvum* (subtype IIaA20G1R1), *C. equi* (VIaA15G3, VIaA11G2), *C. hominis* (IkA15G1) and *C. muris*. *C. equi* and *C. hominis* were reported for the first time in Algerian horses and donkeys, respectively. Genotyping of *G. duodenalis*‐positive samples was unsuccessful due to sequencing failure, likely attributable to low parasite DNA concentrations.

**Conclusions:**

This study reports the first molecular prevalence data for *G. duodenalis* and the first detection of *C. equi* and *C. hominis* in Algerian equines. The zoonotic *C. parvum* subtype IIaA20G1R1, previously detected in Algerian HIV patients, highlights equines as a potential reservoir. Expanded One Health surveillance with longitudinal and seasonal sampling is recommended.

## Graphical abstract

This cross‐sectional study reports the prevalence and molecular characterisation of *Cryptosporidium* spp. and *Giardia duodenalis* in Algerian equines. *C. equi* and *C. hominis* were detected for the first time in Algerian horses and donkeys, respectively, and equines are identified as potential sources of zoonotic *C. parvum* subtypes.

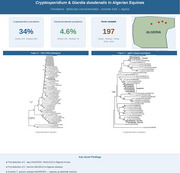

## 1 Introduction

The protozoa *Cryptosporidium* and *Giardia duodenalis* infect the gastrointestinal tract of a wide range of vertebrates, including equines. These pathogens are recognised as a major cause of diarrhoea in farm animals, particularly in young ruminants and in humans, especially in immunocompromised individuals (Santin 2020; Einarsson et al. 2016).

The *Cryptosporidium* genus comprises 49 valid species and more than 120 genotypes (Zikmundová et al. 2025), among them *C. parvum* and *C. hominis*, the most common species infecting humans. *G. duodenalis* comprises eight genetically distinct genotypes (Assemblages A–H), among them, Assemblages A and B are the most common in humans and mammals (Cacciò et al. 2018). Assemblages C and D infect mainly canids, Assemblage F infects felids, while Assemblage E is common in ungulates. These host‐adapted assemblages have been reported less frequently in humans (Ryan and Zahedi 2019). Assemblages G and H have been detected in rodents and pinnipeds, respectively (Ryan and Zahedi 2019).

The epidemiology of cryptosporidiosis in equines remains poorly understood relative to that in ruminants. Prevalence rates ranging from 0% to 38% have been reported worldwide (X. M. Li et al. 2022). *C. equi* (previously known as the horse genotype) and *C. parvum* were commonly identified in horses and donkeys worldwide (Burton et al. 2010; Perrucci et al. 2011; Qi et al. 2015; Inácio et al. 2017; Wang et al. 2020). A wide range of other *Cryptosporidium* spp. have been also detected in equines, including *C. hominis* of particular note, *C. erinacei*, *C. muris*, *C. proliferans*, *C. andersoni*, *C. tyzzeri*, *C. ubiquitum*, *C. felis* and *C. suis* (Laatamna et al. 2013; Guo et al. 2014; N. Li et al. 2014; Liu et al. 2015; Wagnerová et al. 2015; Jian et al. 2016; F. Li et al. 2019; Peng et al. 2025). However, these species are host‐specific to other mammals, and their presence in equine faeces is minimal. Clinical cryptosporidiosis presenting with diarrhoea in foals has been primarily linked to *C. parvum* infections (Grinberg et al. 2008; Perrucci et al. 2011). In contrast, *C. equi* has been identified only rarely in foals with diarrhoea (Caffara et al. 2013). However, the pathogenicity and clinical importance of *C. parvum* and *C. equi* in horses are not yet fully understood. As compared to ruminants, the role of equines as potential hosts of zoonotic subtypes of *C. parvum* and *C. equi* is also not fully understood. Different zoonotic *C. parvum* subtypes belonging to IIa and IId subtype families were identified in horses and donkeys (Jian et al. 2016; F. Li et al. 2019). Furthermore, *C. equi* VIaA15G4 and VIaA11G3 subtypes, frequently reported in equines (Ryan et al. 2003; Jian et al. 2016; F. Li et al. 2019), were recently detected in humans (Zajączkowska et al. 2022; Bujila et al. 2024).

Likewise, data on the epidemiology of giardiasis in equines are similarly limited compared to ruminants. Prevalence rates of *G. duodenalis* in equines ranging between 0% and 73% have been reported worldwide (Mizani et al. 2025). Infection with *G. duodenalis* has been associated with diarrhoea in horses. However, equine giardiasis was often considered an asymptomatic infection (Santín et al. 2013). Four *G. duodenalis* assemblages have been identified in equines, including mainly Assemblages A, B, E and in a rare case Assemblage G. Assemblages A and B were the most frequently reported genotypes, with different distributions from one study to another worldwide (Deng et al. 2017; Mizani et al. 2025).

In Algeria, only two studies have reported prevalence and genotyping of *Cryptosporidium* spp. in horses and donkeys (Laatamna et al. 2013; Laatamna et al. 2015). Furthermore, no Algerian studies have investigated *G. duodenalis* infections in equines. The present cross‐sectional study aims to examine the prevalence and identity of *Cryptosporidium* species/subtypes and *G. duodenalis* assemblages in equines from different regions in central‐northern Algeria.

## 2 Materials and Methods

### 2.1 Study Area, Sample Collection and Microscopic Examination

**FIGURE 1:**
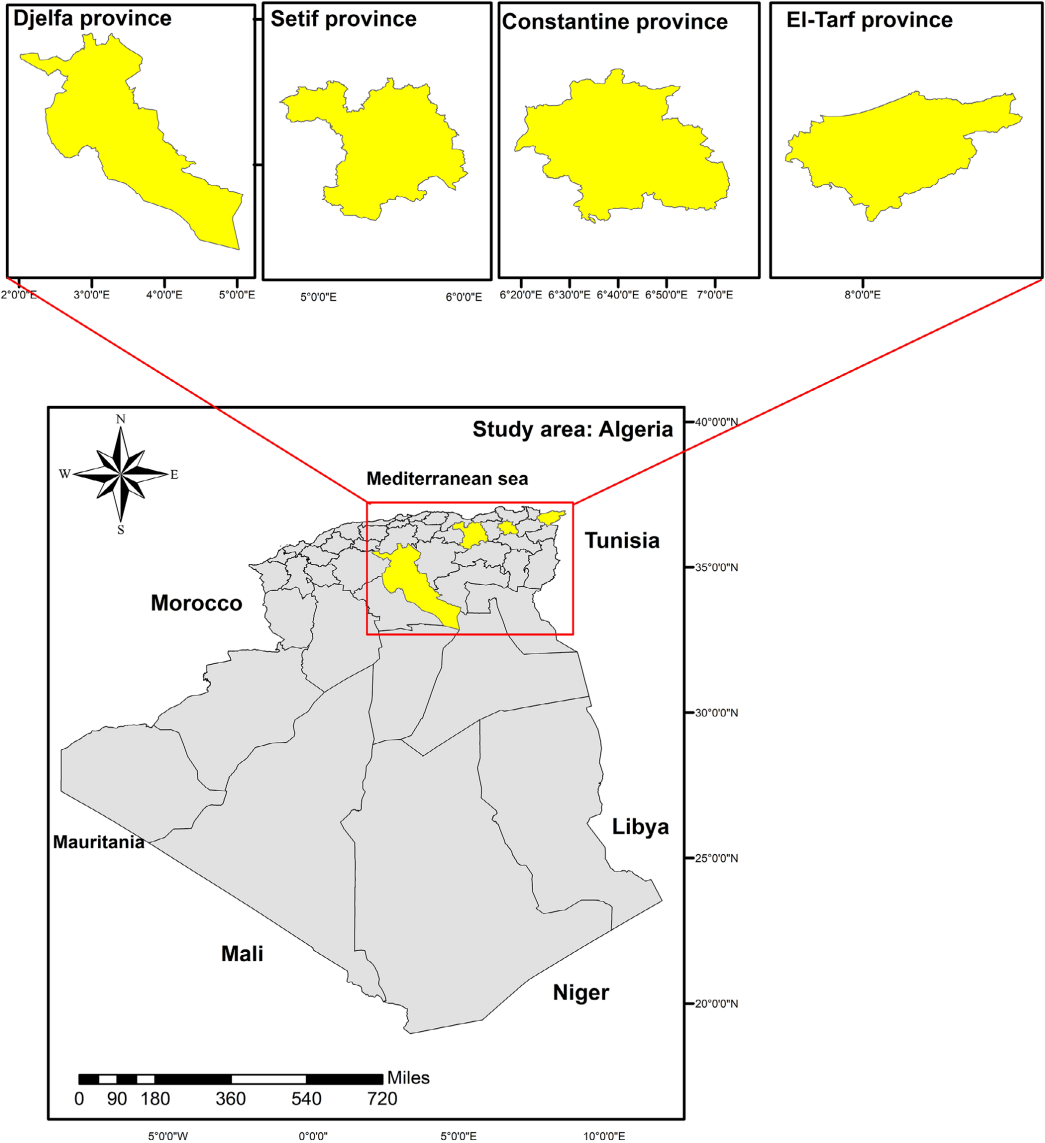
Map of Algeria, showing the four sampling areas in eastern and central of northern Algeria.

From December 2021 to December 2022, a total of 197 faecal samples of different equine hosts, including 104 horses, 89 donkeys, 2 ponies, 1 zebra and 1 mule, were randomly collected from 46 private farms, 2 equestrian centres and 1 zoological park in eastern Algeria (El‐Tarf, Constantine and Setif provinces) and central Algeria (Djelfa province) (Table 1, Figure 1). Horses from private farms and equestrian centres are under different management systems, including extensive, semi‐extensive and intensive breeding. In extensive and semi‐extensive breeding, horses graze for almost the entire year, while in winter, animals are kept in their boxes. Donkeys originated from private farms that graze for almost the entire year, while those sampled from the zoological park of El Taref province were sourced from different rural areas and kept under temporary intensive breeding for later slaughter for the consumption of wild carnivores.

**TABLE 1:** Prevalence rates of *Cryptosporidium* spp. and *Giardia duodenalis* in different hosts of equines in Algeria.

Hosts	Type of farms	No. of farms	No. of screened samples	No. of positive *Cryptosporidium* spp. samples		No. of positive *G. duodenalis* samples	
				Microscopy (%)	PCR (%)	Microscopy (%)	qPCR (%)
Horses	Private farms	34	64	12/64 (18.7)	16/64 (25)	2/64 (3.1)	1/64 (1.6)
	Zoological park	1	1	1/1 (100)	0/1 (0)	0/1 (0.0)	0/1 (0.0)
	Equestrian centres	2	39	15/39 (38.5)	16/39 (41)	2/39 (5.1)	2/39 (5.1)
	Subtotal	37	104	28/104 (27)	32/104 (31)	4/104 (3.8)	3/104 (2.9)
Donkeys	Private farms	12	16	4/16 (25)	4/16 (25)	0 /16 (0.0)	0/16 (0.0)
	Zoological park	1	73	28/73 (37)	30/73 (41)	6/73 (8.2)	6/73 (8.2)
	Subtotal	13	89	32/89 (36)	34/89 (38)	6/89 (7.0)	6/89 (7)
Pony	Zoological park	1	2	0/2 (0)	0/2 (0)	0/2 (0.0)	0/2 (0.0)
Mule	Zoological park	1	1	0/1 (0)	0/1 (0)	0/1 (0.0)	0/1 (0.0)
Zebra	Zoological park	1	1	0/1 (0)	1/1 (100)	0/1 (0.0)	0/1 (0.0)
	Total		197	60/197 (30.5)	67/197 (34.0)	10/197 (5.0)	9/197 (4.6)

Samples were collected directly from the rectum or from the ground after defecation of animals. Faeces of donkeys from the zoological park were collected from the large intestine immediately after slaughter. None of the sampled equines has shown clinical signs of diarrhoea at the time of sampling. For each animal, sampling date, gender, age and farm management were recorded. Each sample was placed in an individual, sterile plastic container and transported in an isothermal box to the laboratory. All samples were examined by microscopy using modified Ziehl–Neelsen staining and Lugol's iodine staining for the presence of *Cryptosporidium* spp. and *G. duodenalis*, respectively. All samples were then preserved in 2.5% potassium dichromate.

### 2.2 Molecular Analysis

#### 2.2.1 DNA Extraction and PCR Analyses

The total DNA was extracted from 250 mg of each faecal sample using a PureLink Microbiome DNA purification kit (Invitrogen, Carlsbad, CA, USA) according to the manufacturer's protocol. The extracted DNA was eluted in 50 µL of elution buffer for DNA concentration. All extracted DNA samples were stored at −20°C until required for screening.

Identification of *Cryptosporidium* species/genotypes and subtypes in DNA samples was performed using nested PCR to amplify fragments of genes encoding the small subunit rRNA (SSU; ∼830 bp) (Xiao et al. 1999; Jiang et al. 2005) and the 60 kDa glycoprotein (*gp60*; ∼830 bp) (Alves et al. 2003). For SSU gene amplification, the primary PCR was performed using the primers F1 (5′‐TTCTAG AGCTAATAC ATGCG ‐3′) and R1 (5′‐CCC ATTTCCTTCGAAACAGGA‐3′). The secondary PCR was performed using the primers F2 (5′‐GGAAGGGTTGTATTTATTAGATAAAG‐3′) and R2 (5′ CTCATAAGGTGCTGAAGGAGTA‐3′). PCR amplification was carried out with an initial denaturation at 95°C for 2 min, followed by 35 cycles of denaturation at 95°C for 30 s, annealing at 50°C/55°C for 45 s and extension at 72°C for 1 min. The final extension step was performed at 72°C for 7 min. For *gp60* gene amplification, the primers AL3531 (5′‐ATAGTCTCCGCTGTATTC‐3′) and AL3535 (5′‐GGAAGGAACGATGTATCT‐3′) were used for the primary PCR and AL3532 (5′‐TCCGCTGTATTCTCAGCC‐3′) and AL3534 (5′‐GCAGAGGAACCAGCATC‐3′) were used for the secondary PCR. Both PCR reactions included an initial denaturation at 94°C for 3 min, followed by 35 cycles of denaturation at 94°C for 45 s, annealing at 50°C for 45 s and extension at 72°C for 1 min, with a final extension step at 72°C for 7 min. Negative (PCR water) and positive controls (DNA of *C. suis* for SSU and *C. hominis* for *gp60*) were included with each PCR run. The PCR amplicons were electrophoresed in 2% agarose gels with 0.2 mg/mL ethidium bromide and visualised under ultraviolet light.

Detection of *G. duodenalis* was performed using specific probe‐based quantitative real‐time PCR targeting the SSU gene (product size: ∼62 bp), as previously described (Maxamhud et al. 2023). Each PCR reaction consisted of a total volume of 20 µL containing 10 µL of 1 × Luna Universal Probe qPCR Master Mix (New England Biolabs, MA, USA), 0.8 µL of 0.4 µM *G. duodenalis*‐specific primers; *Giardia*‐80F (5′‐GACGGCTCAGGACAACGGTT‐3′) and *Giardia*‐127R (5´‐TTGCCAGCGGTGTCCG‐3′), 0.4 µL of 0.2 µM *G. duodenalis*‐specific probe (FAM‐5′‐CCCGCGGCGGTCCCTGCTAG‐3′‐black hole quencher), 1 µL of 0.5 µg/µL bovine serum Albumin (BSA opposing the effect of PCR inhibitors in faeces) (Promega Madison, WI, USA), 5 µL of nuclease‐free H_2_O (Promega Madison, WI, USA) and 2 µL of extracted DNA. PCR conditions were as follows: An initial denaturation step at 95°C for 2 min, followed by 50 cycles of 95°C for 15 s, annealing at 57°C for 30 s and a final elongation step at 72°C for 30 s. The qPCR positive samples were furthermore genotyped through nested PCR protocols targeting two loci, including the triosephosphate isomerase (*tpi*) (Sulaiman et al. 2003), and *β* giardin (*bg*) (Cacciò et al. 2002). In each PCR run, a positive sample of *G. duodenalis* was used as a positive control and PCR water as a negative control. The PCR amplicons were also electrophoresed in 1.5% agarose gels with 0.2 mg/mL ethidium bromide and visualised under ultraviolet light.

#### 2.2.2 Sequencing and Sequences Analyses

All positive PCR products were purified using the Thermo Scientific GeneJET Gel Extraction Kit (Thermo Fisher Scientific, CA, USA) according to the manufacturer's instructions and subsequently sequenced bidirectionally on an ABI 3730XL sequencer (Applied Biosystems, Foster City, CA, USA). Obtained nucleotide sequences were assembled using ChromasPro 2.2.0 (Technelysium, Pty, Ltd.) and edited using BioEdit 7.0.5.3. The identity of *Cryptosporidium* spp. was first determined by comparison of nucleotide sequences from the present study with each other and with GenBank reference sequences using the Basic Local Alignment Search Tool (BLAST) (http://blast.ncbi.nlm.nih.gov/Blast.cgi). The sequences of the SSU and *gp60* genes obtained in this study were aligned with reference sequences using the MAFFT Version 7 online server, employing the Q‐INS‐i algorithm for the SSU sequence and the L‐INS‐i algorithm for the *gp*60 sequence (http://mafft.cbrc.jp/alignment/server/) (Katoh and Standley 2013). Alignment adjustments were made manually to remove artificial gaps using BioEdit 7.0.5.3. Phylogenetic relationships of sequences were inferred using neighbour‐joining (NJ) methods (Saitou and Nei 1987), based on the Kimura 2‐parameter (K2P) distances model (Kimura 1980) with pairwise deletions. Phylogenetic trees were constructed using MEGA7 v.7.0. (Kumar et al. 2016). The reliability of branches in trees was assessed using the bootstrap analysis with 1000 pseudo‐replicates. Bootstrap values above 50% were reported.

Generated *Cryptosporidium* sequences from the present study were deposited in GenBank under the following accession numbers: PV667525–PV667534 for SSU rRNA gene and PV684234–PV684242 for *gp60* gene.

#### 2.2.3 Statistical Analysis

Statistical analysis was performed using the PAST software 4.17c. Tests of risk ratio and *χ* ^2^ were used to assess differences in the frequency (prevalence rates) of *Cryptosporidium* spp. and *G. duodenalis* by epidemiological variables, including equine host, gender, age and farm management. *p* ≤ 0.05 (two‐tailed) was considered statistically significant.

## 3 Results

### 3.1 Prevalence of *Cryptosporidium* spp. and *G. duodenalis*

Overall, microscopic and PCR analyses revealed prevalences of 30.5% and 34% for *Cryptosporidium* spp. and 5% and 4.6% for *G. duodenalis*, respectively (Table 1). In horses, the prevalence of *Cryptosporidium* was 27% and 31% by microscopy and PCR, respectively. *G. duodenalis* was detected in 3.8% and 2.9% of screened horses by both methods, respectively. In donkeys, *Cryptosporidium* prevalence was 36% and 38% by microscopy and PCR, respectively, while *G. duodenalis* was detected in 7% of screened donkeys by both methods. The samples analysed from pony and mule were negative for *Cryptosporidium* and *G. duodenalis* by both techniques, while the one sample from zebra was positive for *Cryptosporidium* by PCR analysis (Table 1).

All statistical analyses were conducted to assess prevalence rates based on molecular results. Detection of *Cryptosporidium* spp. did not differ significantly by host, farm management and age of equines (*p* > 0.05), while male equines had a significantly higher infection rate than females (Table 2). The prevalence of *G. duodenalis* did not differ significantly across all epidemiological variables, including host, age, sex and farm management (Table 3).

**TABLE 2:** Prevalence of *Cryptosporidium* spp. by host, age, gender and farm management of equines.

Variable	No. of positive samples	No. of negative samples	Prevalence (%)	*p* value	Risk difference	95% confidence of risk difference	*χ* ^2^
Host	**66**	**127**	**34.2 (66/193** ^a^)	0.280	−0.074	[−0.0600; 0.2080]	1.180
Horses	32	72	30.8 (32/104)				
Donkeys	34	55	38.2 (34/89)				
Farm management	**67**	**130**	**34.0 (67/197** ^b^)	1.447	−0.002	[−0.1703; 0.1654]	0.0008
Extensive	41	88	31.8 (41/129)				
Semi‐extensive	13	17	43.3 (13/30)				
Intensive	13	25	34.2 (13/38)				
Age	**67**	**130**	**34.0 (67/197** ^b^)	0.216	−0.150	[−0.3449; 0.0330]	1.940
≤ 1year	4	16	20.0 (4/20)				
> 1 year	63	114	35.6 (63/177)				
Sex	**67**	**130**	**34.0 (67/197** ^b^)	0.026	0.150	[0.0185; 0.2818]	4.930
Males	39	54	41.9 (39/93)				
Females	28	76	26.9 (28/104)				

*Note*: Here, 95% confidence interval on risk difference (Pearson's *χ* ^2^).

^a^ Zebra, pony and mule were not included in the host variable due to low sample size.

^b^ Zebra, pony and mule were included.

**TABLE 3:** Prevalence of *Giardia duodenalis* by host, age, gender and farm management of equines.

Variable	No. of positive samples	No. of negative samples	Prevalence (%)	*p* value	Risk difference	95% confidence of risk difference	*χ* ^2^
Host	**9**	**184**	**4.7 (9/193** ^a^)	0.210	0.0390	[0.0230; 0.1000]	1.60
Horses	3	101	2.9 (3/104)				
Donkeys	6	83	6.7 (6/89)				
Farm management	9	**188**	**4.6 (9/197** ^b^)	0.330	−0.03578	[−0.1294; 0.0537]	0.92
Extensive	5	124	3.9 (5/129)				
Semi‐extensive	1	29	3.3 (1/30)				
Intensive	3	35	7.9 (3/38)				
Age	**9**	**188**	**4.6 (9/197** ^b^)	0.301	−0.05084	[−0.0830; −0.0180]	1.06
≤ 1year	0	20	0.0 (0/20)				
> 1 year	9	168	5.0 (9/177)				
Sex	**9**	**188**	4.6 (9/197^b^)	0.607	0.01530	[−0.0430; 0.0740]	0.26
Males	5	88	5.4 (5/93)				
Females	4	100	3.8 (4/104)				

*Note*: The 95% confidence interval on risk difference (Pearson's *χ* ^2^).

^a^ Zebra, pony and mule were not included in the host variable due to low sample size.

^b^ Zebra, pony and mule were included.

### 3.2 Genotyping of *Cryptosporidium* spp. and *G. duodenalis*

**FIGURE 2:**
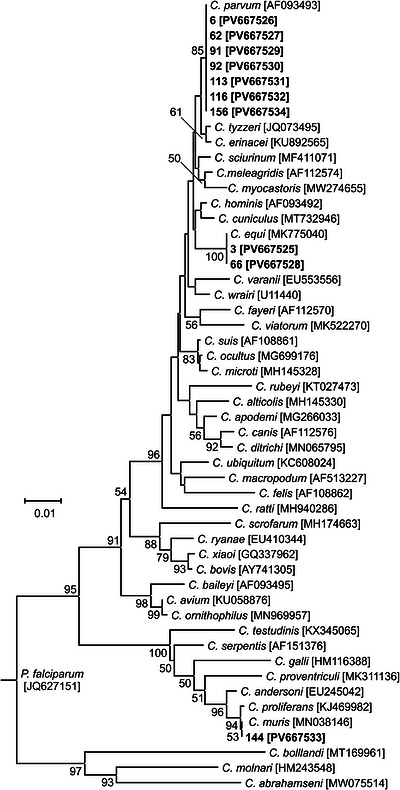
Phylogenetic relationship among *Cryptosporidium* species, including isolates identified in the present study (highlighted), inferred based on partial sequences of the small subunit of the ribosomal gene using the neighbour‐joining method. Numbers at the nodes represent bootstrap values for nodes gaining more than 50% support. The scale bar is shown in the tree.

**FIGURE 3:**
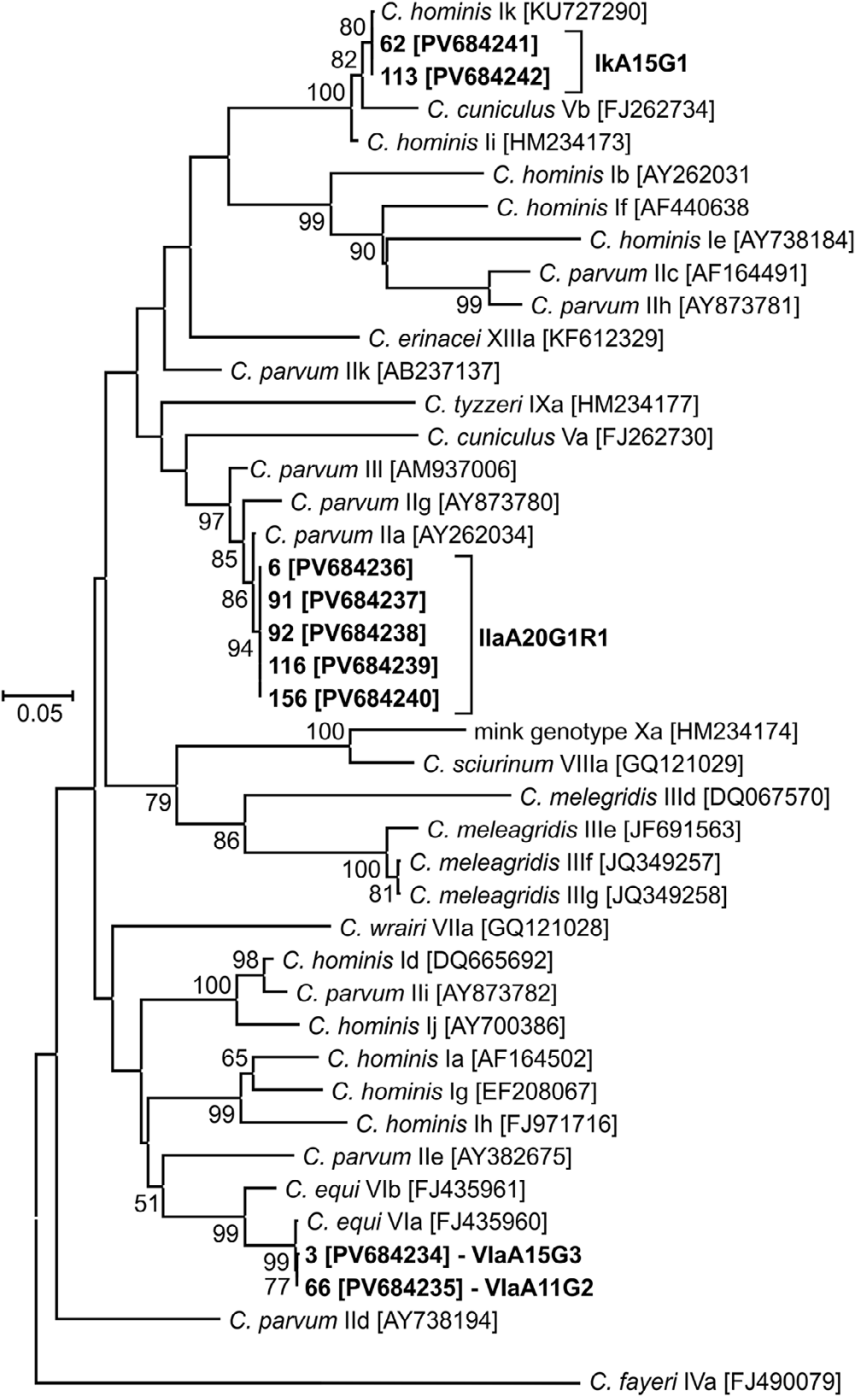
Phylogenetic relationship among subtypes of *Cryptosporidium* spp., including subtypes identified in the present study (highlighted), inferred based on partial sequences of the *gp*60 gene using the neighbour‐joining method. Numbers at the nodes represent bootstrap values for nodes gaining more than 50% support. The scale bar is shown in the tree.

Among PCR‐positive *Cryptosporidium* samples, 10 isolates were successfully genotyped and subtyped. These samples consisted of nine horses (isolates no. 3, 6, 66, 91, 92, 113, 116, 144, 156) and one donkey (isolate no. 62). Partial sequences of seven, two and one samples showed 100% identity with *C. parvum* (accession number: AF093493), *C. equi* (MK775040) and *C. muris* (MN038146) reference sequences from GenBank, respectively. Based on the phylogenetic analysis of the SSU gene, seven samples (horses no. 6, 91, 92, 113, 116, 156 and donkey no. 62) were clustered with the *C. parvum* reference sequence. Two samples (horses no. 3 and 66) were clustered with *C. equi* reference sequence, while one sample (horse no. 144) was clustered with *C. muris* reference sequence (Figure 2). Of the seven *C. parvum* samples, the phylogenetic analysis of the *gp60* gene, identified five isolates (horses no. 6, 91, 92, 116 and 156) as subtype IIaA20G1R1, while two isolates (horse no. 113 and donkey no. 62) clustered with the *C. hominis* subtype IkA15G1 (Figure 3). Therefore, horse no. 113 and donkey no. 62 showed a mixed infection with *C. parvum* and *C. hominis*. *C. equi* samples belonged to two different subtypes, namely VIaA15G3 and VIaA11G2 (Figure 3).

Nine samples were positive for *G. duodenalis* by qPCR, with *C*_t_ values ranging from 31.26 to 43.96 (the positivity threshold applied was *C*_t_ < 45). High *C_t_* values close to the limit of detection likely reflect low DNA amounts in samples. All these *G. duodenalis* samples were successfully amplified by nested PCR of the *tpi* and *bg* genes, but could not be genotyped due to sequencing failure of PCR products for both genes.

## 4 Discussion

Limited data are available on cryptosporidiosis in equines in Algeria, and no prior data exist on giardiasis in Algeria. Equine *Cryptosporidium* infection has been reported in only two Algerian studies (Laatamna et al. 2013; Laatamna et al. 2015). The present study reports the first preliminary data on the prevalence of *G. duodenalis* in horses and donkeys and the first occurrence of *Cryptosporidium* spp. in a zebra from Algeria.

Overall, the 34% prevalence of *Cryptosporidium* in equines observed in this study was comparable to some studies (Caffara et al. 2013; Guo et al. 2014). However, it was higher than the prevalence reported in previous Algerian studies (Laatamna et al. 2013, Laatamna et al. 2015) and in most other studies worldwide (X. M. Li et al. 2022). The low prevalence of *G. duodenalis* in horses in this study aligns with many previous studies (0.5%–4.0%) (Atwill et al. 2000; De Souza et al. 2009; Qi et al. 2015; F. Li et al. 2020). Conversely, several studies worldwide documented higher prevalences (up to 73%) of *G. duodenalis* in horses (Mizani et al. 2025). Some studies from China reported relatively higher prevalences (11.5%–15.5%) of *G. duodenalis* in donkeys compared to our results (Zhang et al. 2017; F. Li et al. 2020).

The prevalence of *Cryptosporidium* did not vary significantly by host, age or farm management of equines. Discordant results between microscopy and PCR were noted in equines from the zoological park (one horse and one zebra), likely attributable to the very small sample size from this setting and other factors such as low oocyst load limiting the microscopic detection, morphological misidentification or presence of PCR inhibitors in faeces. Overall, X. M. Li et al. (2022) documented a higher prevalence in equines under 1‐year‐old as compared to those over 1‐year‐old. Foals were found to be more infected than adults (Xiao and Herd 1994; Veronesi et al. 2010), while no association between *Cryptosporidium* prevalence and age of horses was found (Laatamna et al. 2015; Wagnerová et al. 2015). In contrast to our study, the prevalence of *Cryptosporidium* in equine females did not differ from that in males (X. M. Li et al. 2022). Similarly to our study, no relationship was found between *Cryptosporidium* detection and farm management of equines (Laatamna et al. 2015). High infection rates within farms with high equine density might be observed, as suggested by X. M. Li et al. (2022). The influence of breeding management on variations in infection rates is difficult to demonstrate, as most reported prevalences are low among equine populations.

The prevalence of *G. duodenalis* was not significantly linked to age, sex or farm management of equines. Similar findings on age and sex of horses were reported (Atwill et al. 2000; Santín et al. 2013; Qi et al. 2020). In contrast, a higher infection rate was observed in foals compared to adults (Veronesi et al. 2010). Significant differences in *G. duodenalis* prevalence among various equine clubs were observed, suggesting that the different management practices within these clubs may contribute to variations in prevalence (Qi et al. 2020).

Compared to ruminants, factors influencing the variation in prevalence of cryptosporidiosis and giardiasis in equines are not fully understood, particularly host‐related factors (e.g., age) and husbandry practices. In this study, the prevalence of both parasites showed no age association; such findings remain inconclusive due to the small sample size for the younger group. Nonetheless study design, sample size and diagnostic techniques used for detecting both parasites could also influence the variation in prevalence rates.

The present study identified *C. parvum*, *C. equi*, *C. hominis* and *C. muris* in horses, and *C. parvum* and *C. hominis* in donkeys. Previous Algerian studies reported *C. erinacei*, *C. parvum*, *C. hominis* and *C. muris* in horses, and *C. parvum* and *C. proliferans* in donkeys (Laatamna et al. 2013, Laatamna et al. 2015). This study revealed, for the first time, the occurrence of *C. equi* in horses and *C. hominis* in donkeys. Most molecular studies worldwide have reported the presence of *C. parvum*, *C. equi* and *C. hominis* in horses and donkeys (Table S1). The occurrence of non‐specific *Cryptosporidium* species in equines may be due to passive infection, incidental environmental contamination or cross‐transmission (Liu et al. 2015; Wagnerová et al. 2015). In addition, there might be a potential susceptibility of certain equids to these non‐specific species, although the underlying factors of which remain unknown.

In agreement with this study, *C. hominis* has been commonly detected in horses and donkeys from various countries, especially China (Laatamna et al. 2015; Jian et al. 2016; Deng et al. 2017; F. Li et al. 2019; Wang et al. 2020). Various subtype families of *C. hominis*, including the Ik subtype family, have been shown to exhibit host‐adaptation (Feng et al. 2018). The IkA15G1 subtype, detected in donkeys in this study, was previously identified in horses in Algeria (Laatamna et al. 2015). Other subtypes of the Ik subtype family have been identified in horses and donkeys from other countries (Table S1). Ik displayed divergent genetic nature and was considered an equine‐adapted *C. hominis* subtype family (Feng et al. 2018). Nonetheless, this subtype family has also been detected in a cow from the United Arab Emirates (Procter et al. 2024), a Bactrian camel from China (Cao et al. 2020) and in two human patients in Sweden (Lebbad et al. 2018). More evidence is needed to determine the full host range and the zoonotic potential of the Ik *C. hominis* subtype family.

All *C. parvum* isolates detected in this study belonged to the IIaA20G1R1 subtype family. Various subtypes belonging to zoonotic *C. parvum* subtype families IIa (e.g., the major hyper‐transmissible zoonotic subtype IIaA15G2R1) and IId have been detected in equines (Table S1). In Algeria, different *C. parvum* subtypes of IIa and IId, including the IIaA20G1R1 detected in horses in this study, have been detected in HIV patients (Semmani et al. 2023). Consequently, equines could be a potential source of zoonotic *C. parvum* subtypes for humans.

In this study, *C. equi* was subtyped for the first time in Algerian horses. Two subtypes, A15G3 and A11G2, belonging to the VIa subtype family, were detected. The VIa seems to be an equine adapted subtype family, which was mainly identified in horses and donkeys, with the VIaA15G4 being the most reported subtype worldwide (Huang et al. 2023; Table S1). Recently, VIaA15G4 and VIaA11G3 subtypes were detected in two human patients from Poland and Sweden, respectively (Zajączkowska et al. 2022; Bujila et al. 2024). In Poland, the same subtype VIaA15G4 was detected in a horse the patient had frequently ridden, suggesting a possible direct transmission of cryptosporidiosis (Zajączkowska et al. 2022). Further investigations are needed, particularly in humans living in frequent contact with equines from rural areas, to demonstrate the role of equines as an important source of *C. equi* subtype VIa family. Other known subtype families of *C. equi*, including VIb (VIbA13 subtype), VIc (VIcA16) and VId (VIdA10G1), have, to our knowledge, not yet been identified in equines and have been detected in patients with cryptosporidiosis from United Kingdom, United States of America and Sweden (Robinson et al. 2008; Xiao et al. 2009; Lebbad et al. 2021; Bujila et al. 2024).

Although the present study reports for the first time the prevalence of *G. duodenalis* in horses and donkeys from Algeria using PCR analyses, the inability to sequence PCR products for genotyping, likely due to low parasite DNA concentrations, represents the limitation of our study. In equines, the Assemblage B was the most reported genotype from various studies (Santín et al. 2013; Deng et al. 2017; Zhang et al. 2017; Qi et al. 2020), while Assemblage A was the dominant one from some other studies (Mukbel et al. 2017; Demircan et al. 2019). The Assemblage E (common in hoofed animals) was globally reported less frequently (Mizani et al. 2025), while it was the only genotype identified in horses in some studies (Veronesi et al. 2010).

## 5 Conclusion

The present study emphasised the role of equines as a potential source of zoonotic *C. parvum* and demonstrated the circulation of *C. hominis* and *C. equi* within equine populations. Further studies are needed to elucidate the distribution and transmission dynamics of these pathogens in horses and donkeys, as well as to assess their public health significance. In addition, further molecular studies are required to understand the distribution of *G. duodenalis* assemblages in equines in Algeria.

## Author Contributions

**Nassiba Reghaissia**: conceptualisation, investigation, methodology, visualisation, data curation. **Yaseen Majid Salman Al‐Adilee**: investigation, methodology, visualisation, data curation. **AbdElkarim Laatamna**: writing – original draft, writing – review and editing, supervision. **Abdeldjalil Dahmane**: investigation. **Amine Berghiche**: formal analysis. **Houssem Samari**: investigation. **Samir Ansel**: investigation. **Yacine Laadjailia**: investigation. **Eleni Gentekaki**: writing – review and editing. **Martin Kváč**: methodology, visualisation, formal analysis, writing – review and editing. **Nikola Holubová**: methodology, formal analysis. **Anastasios D. Tsaousis**: project administration, supervision, resources, writing – review and editing.

## Funding

This study was funded by Czech Science Foundation, Grant/Award Number: 21–23773S.

## Ethics Statement

The ethics committee of the Laboratory of Exploration and Valorisation of Steppic Ecosystems from the Faculty of Nature and Life Sciences, Ziane Achour University‐Djelfa, Algeria, approved the collection of samples from equines in this study (003 EVES/FSNV/2021).

## Conflicts of Interest

The authors declare no conflicts of interest.

## Supporting information




**Supplement Table 1**: Distribution of Cryptosporidium species/genotypes and subtypes in horses and donkeys worldwide.

## Data Availability

The data that support the findings of this study are available from the corresponding authors upon reasonable request.
