# Supplementary material for: Prevalence and Molecular Characterisation of Cryptosporidium spp. and Giardia duodenalis in Equines in Algeria
Source: Vet Med Sci. 2026 Jul 15;12(4):e71083. doi: 10.1002/vms3.71083 (PMC13371905; doi:10.1002/vms3.71083)
Supplement: Supplementary file 1 — Supplement Table 1: Distribution of Cryptosporidium species/genotypes and subtypes in horses and donkeys worldwide. [file VMS3-12-e71083-s001.docx]

| Country | Equine species | N° of isolates | Species/genotypes of Cryptosporidium (n) | Subtypes | References |
| --- | --- | --- | --- | --- | --- |
| Algeria | Horses | 9 | *C. parvum* (6) | IIaA20G1R1 | This study |
|  | Horses |  | *C. equi* (2) | VIaA15G3, VIaA11G2 |  |
|  | Horses |  | *C. hominis* (1) | IKA15G1 |  |
|  | Horses |  | *C. muris* (1) |  |  |
|  | Donkey | 1 | *C. parvum* (1) | Not subtyped |  |
|  | Donkey |  | *C. hominis* (1) | IKA15G1 |  |
| Algeria | Horses | 4 | *C. Erinacei (4)* | XIIIa A22R9 | (Laatamna et al., 2013) |
| Algeria | Horses | 5 | *C. parvum* (3) | IIaA16G1R1 | (Laatamna et al., 2015) |
|  | Horses |  | *C. hominis* (1) | IkA15G1 |  |
|  | Horses |  | *C. muris* (1) |  |  |
|  | Donkey | 2 | *C. parvum* (1) | IIaA16G1R1 |  |
|  | Donkey |  | *C. proliferans* (1)  Supplement table 1. Distribution of *Cryptosporidium* species/genotypes and subtypes in horses and donkeys worldwide. |  |  |
| USA | Horses | 7 | *C. equi* (7) | VIaA14G2 | (Burton et al., 2010) |
| USA | Mustangs | 3 | *C. parvum* (3) | IIaA17G2R1 | (Wagnerová et al., 2016) |
|  | Ponies | 17 | *C. parvum* (17) | IIaA15G2R1, IIaA13G2R1 |  |
| Italy | Horses | 12 | *C. parvum* (12) | Not subtyped | (Veronesi et al., 2010) |
| Italy | Horses | 4 | *C. parvum* (4) | Not subtyped | (Perrucci et al., 2011) |
| Italy | Horses | 12 | *C. parvum* (3) | Not subtyped | (Caffara et al., 2013) |
|  | Horses |  | *C. equi* (9) | VIaA15G4 |  |
| Italy | Horses | 14 | *C. parvum* (4) | IIdA21G1, IIdA22G1, IIaA23R1 | (Galuppi et al. 2015) |
|  | Horses |  | *C. equi* (10) | VIaA15G4 |  |
|  | Horses |  | *C. parvum*+ *C. equi* (4) |  |  |
| Italy | Horses | 2 | *C. parvum* (2) | IIdA23G1 | (Galuppi et al. 2016) |
| New Zealand | Horses | 3 | *C. parvum* (3) | Not subtyped | (Grinberg et al., 2003) |
| New Zealand | Horses | 9 | *C. parvum* (9) | IIaA18G3R1 | (Grinberg et al., 2008) |
| Czech Republic | Horses | 1 | *C. parvum* (1) | IIaA15G2R1 | (Wagnerová et al., 2015) |
|  | Horses | 1 | *C. equi* (1) | VIaA15G4 |  |
|  | Horses | 7 | *C. muris* (7) | M1, M7, M4 |  |
|  | Horses | 1 | *C. tyzzeri* (1) | IXbA22R9 |  |
| Poland | Horses | 2 | *C. muris* (2) | M1, M4 | (Wagnerová et al., 2015) |
| Czech Republic | Horses | 3 | *C. parvum* (3) | not subtyped | (Hajdušek et al., 2004) |
| Germany | Horses | 1 | *C. parvum* (1) | not subtyped | (Imhasly et al., 2009) |
| Belgium and Greece | Horses | 2 | *C. equi* (2) | not subtyped | (Kostopoulou et al., 2015) |
| Czech Republic | Przewalski’s wild horse | 1 | *C. equi* (1) | VIaA11G3 | (Ryan et al., 2003) |
| Ireland | Horses | 7 | *C. parvum* (3) | not subtyped | (Mirhashemi et al., 2015) |
|  | Horses |  | *C. bovis* (1) |  |  |
|  | Horses |  | *C. andersoni* (1) |  |  |
|  | Horses |  | *C. ryanae* (4) |  |  |
| Irland | Horses | 23 | *C. parvum* | IIjA15G4 | (Mirhashemi et al., 2016) |
|  | Horses |  | *C. equi* | Not subtyped |  |
|  | Horses |  | *C. bovis* |  |  |
|  | Horses |  | *C. ryanae* |  |  |
|  | Horses |  | *C. bovis*/*C. xiaoi* |  |  |
|  | Horses |  | *C. andersoni/C. bovis* |  |  |
|  | Horses |  | *Cryptosporidium pig (not genotyped*  inappropriately) |  |  |
| Norvay | Horses | 8 | *C. parvum* (8 ) | Not subtyped | (Paruch and Paruch 2022) |
| UK (wales) | Horses | 2 | *C. parvum (2)* | Not subtyped | (Chalmers et al., 2005) |
| UK | Horses | 1 | *C. ubiquitum* | XIIa | (Li et al., 2014) |
| Iberian Peninsula | wild ponies | 7 | *C. parvum* (7) | IIaA15G2R1 (2), IIaA16G3R1 | (Couso-Pérez et al., 2020) |
| Jordan | Horses | 3 | *C. parvum* (1) | Not subtyped | (Hijjawi et al., 2016) |
|  | Horses |  | Novel (not named) (2) | Not subtyped |  |
| China | Horses | 11 | *C. parvum* (7) | IIdA14G1, IIdA15G1 | (Xu et al., 2023) |
|  | Horses |  | *C. hominis* ( 4) | IkA18G1 |  |
| China | Donkey | 16 | *C. parvum* (1) | IIdA15G1 | (Wang et al., 2020) |
|  | Donkey |  | *C. equi* (2) | VIaA15G4 |  |
|  | Donkey |  | *C. hominis* (13) | IkA16, IkA16G1 |  |
| China | Horses | 7 | *C. equi* (7) | VIaA15G4 | (Qi et al., 2015) |
| China | Horses | 4 | *C. suis* (4) | Not subtyped | (Peng et al., 2025) |
| China | Horses | 6 | *C. hominis* (2) | IdA15 (renamed as IoA15) | (Deng et al., 2017) |
|  | Horses |  | *C. andersoni* (4) | A6, A5, A2, and A1 |  |
| China | Horses | 5 | *C. parvum* (4) | IIdA19G1 | (Jian et al., 2016) |
|  | Horses |  | *C. hominis* (1) | IkA16G1 |  |
|  | Donkey | 82 | *C. parvum* (18) | IIdA19G1 |  |
|  | Donkey |  | *C. equi* (4) | VIaA15G4,VIaA11G3 |  |
|  | Donkey |  | *C. hominis* (60) | IkA16G1, IkA16 |  |
| China | Horses | 10 | *C. parvum* (5) | IIdA19G1 | (Li et al., 2019) |
|  | Horses |  | *C. equi* (5) | VIaA15G4 |  |
|  | Donkey | 80 | *C. hominis* (75) | IkA16G1 |  |
|  | Donkey |  | *C. equi* (4) | VIaA15G4 |  |
|  | Donkey |  | *mink genotype-like* (1) |  |  |
| China | Horses | 2 | *C. andersoni* (2) | A4, A4, A4, and A1 | (Liu et al., 2015) |
| Taiwan | Horses | 33 | *C. parvum* (31) | Not subtyped | (Guo et al., 2014) |
|  | Horses |  | *C. felis* (2) | Not subtyped |  |
| Brazil | Horses | 5 | *C. parvum* (3) | IIaA15G2R1 (2), IIaA18G3R1 | (Inácio et al., 2017) |
|  | Horses |  | *C. hominis* (2) | IkA20G1 |  |
| Brazil | Horses | 8 | *C. parvum* (7) | IIaA15G2R1 | (Ribeiro  et al., 2022) |
|  | Horses |  | *C. felis* (1) | Not subtyped |  |
| United Arab Emirates | Horses | 95 | *C. parvum* (90) | IIa, IId | (Procter et al., 2024) |
|  | Horses |  | *C. hominis* (3) | Ik |  |
|  | Horses |  | *C. equi* (4) | VIa |  |
